# Development and validation of self-management scale for tuberculosis patients

**DOI:** 10.1186/s12879-022-07483-3

**Published:** 2022-05-27

**Authors:** Jin Li, Liwen Zhang, Jiani Zhou, Geng Wang, Rui Zhang, Jiaqing Liu, Shili Liu, Yong Chen, Song Yang, Quan Yuan, Ying Li

**Affiliations:** 1 Department of Social Medicine and Health Service Management, Army Medical University (Third Military Medical University), Chongqing, China; 2 Chongqing Public Health Medical Center, Chongqing, China

**Keywords:** Instrument development, Self-management, Tuberculosis

## Abstract

**Background:**

Tuberculosis remains a major threat to global public health. Regarding its control, directly observed therapy is not suitable as a global strategy for all tuberculosis patients. Self-management may be an important patient-centered tuberculosis case management supplement to directly observed therapy. However, there is currently no well-established instrument for measuring the self-management of tuberculosis patients. This study aimed to develop and validate a self-management scale for tuberculosis patients.

**Methods:**

We developed an initial scale based on the tuberculosis health promotion indicators framework developed by our research group. After item analysis and two rounds of exploratory factor analysis, a final version of the scale was developed. A survey of 462 tuberculosis patients was conducted to develop and validate this scale. Cronbach’s α and intraclass correlation coefficients were used to assess reliability, and Pearson’s correlation coefficients were used to evaluate content validity. Fit indices, convergent validity, and discriminant validity were evaluated using confirmatory factor analysis to determine the construct validity of the scale.

**Results:**

The scale was composed of 17 items in three dimensions (“adherence to treatment behavior,” “transmission prevention behavior,” and “supportive therapy behavior”). These three dimensions explained 76.60% of the variance. Cronbach’s α of the scale was 0.905, and the intraclass correlation coefficient was 0.897. Additionally, Pearson’s correlation analysis showed that each item was strongly correlated with the dimension to which it belonged (r = 0.849–0.915, *p* < 0.01). Most fit indices (Comparative Fit Index, Normed Fit Index, Incremental Fit Index, Goodness of fit index) reached the recommended threshold, and the average variance extracted values of the three dimensions were higher than 0.5. The values of the square root of the average variance extracted within each dimension were greater than the correlation between dimensions, and all heterotrait–monotrait values were below 0.85.

**Conclusions:**

The self-management scale for tuberculosis patient demonstrated good reliability and validity and could be used as an instrument to evaluate the self-management of patients. Additionally, it could be used to develop evidence-based self-management interventions and evaluate those interventions.

## Background

Tuberculosis (TB) is one of the most serious problems threatening global health today [1]. In 2020, approximately 10 million new cases and 1.5 million deaths due to TB were reported globally [1]. To prevent the development of drug resistance, compliance with anti-TB treatment is essential. However, patient compliance is a challenge, as treatment is typically long-term (at least 6 months). The World Health Organization (WHO) recommends directly observed therapy, short-course (DOTS) to control TB [2], where patients ingest medications under the direct observation of medical workers as a case management strategy to improve compliance.

According to WHO, China’s National Tuberculosis Control Program is one of the most successful DOTS programs in the world [3]. However, the results of a survey found more than one-third of TB patients in Chongqing were never supervised by any healthcare workers [4]. A meta-analysis [5] indicated that only 20% of patients with TB were covered by Directly observed therapy (DOT) in China, which was much lower than reported by official statistics. DOT requires that patients either go to a clinic or have DOT workers come to their homes to observe the ingestion of medication [6]. In this way, DOT remains time-consuming, resource-intensive, difficult to achieve over distances, and represents the largest single cost of TB treatment [7]. Furthermore, DOT is controversial due to challenges associated with autonomy and leaving patients as passive recipients of treatment [8]. Individual studies have described DOT as intrusive and disempowering for patients [7, 9]. Therefore, reasons for patients’ failure to attend DOT mainly include a lack of primary medical personnel, poor transport access, and patients’ concerns about privacy, autonomy, dignity, and integrity [10–12]. Therefore, WHO recommended the people-centered care strategies in 2014, which encourage patient self-management and community engagement when medically feasible [13, 14]. Patient-centered care and supervision must be carried out in a context-specific and patient-sensitive manner to treatment interruption.

There is no standard definition of self-management. Barlow et al. [15] depicted self-management as “An individual’s ability to manage the symptoms, treatment, physical, psychosocial consequences, and lifestyle changes inherent in living with a chronic disease”. Lorig and Holman [16] defined self-management as “learning and practicing skills necessary to maintain an active and emotionally satisfying life in the face of a chronic disease”. In this study, we defined self-management as the ability to actively manage disease in daily life, change lifestyles closely related to the disease, and effectively cope with the physical and mental effects of the disease. Effective patient-centered TB case management emphasizes the patient needs [17] and requires individually tailored management strategies among TB patients, rather than universally applying DOT. Furthermore, self-management of symptoms, treatment, and physical and psychological problems [15, 18] can significantly improve medication adherence in patients with chronic illness [19, 20], help establish healthy behaviors [21, 22], and promote treatment outcomes [23, 24]. More than half of TB or multidrug-resistant TB (MDR-TB) patients self-administer treatments [25, 26]. Therefore, self-management of TB patients (SMTP) is important and could improve treatment outcomes if sufficient knowledge and skills are promoted [27, 28]. Moreover, SMTP may be a useful supplement for patients who are reluctant to accept or with limited access to DOT. However, little research has been conducted on SMTP, with only a few studies have discussed the definition of [29, 30], factors associated with [31–33], and interventions using SMTP [34, 35]. However, understanding and developing an instrument to measure SMTP before intervention is crucial.

SMTP measurement is the foundation for decision-making regarding SMTP intervention delivery and evaluation. Two studies explored tools related to measuring the behaviors of patients with TB, one of which explored the scale of health self-management assessments for elderly patients with TB. The study primarily evaluated the knowledge, attitude, and behaviors (health responsibility, psychology, physical activities, and nutrition) of those patients [36]. The second study developed a self-discipline scale for TB patients treated at home, which evaluated factors including access to information, compliance, isolation control, and daily life management [37]. However, neither study proposed an instrument for evaluating the key behaviors related to SMTP, such as prevention and treatment. Therefore, to our knowledge, no study has yet developed an instrument to measure SMTP.

To address these gaps in knowledge, this study aimed to develop and validate a self-management scale for TB patients (SMSTP), which could be used to measure SMTP and evaluate the effectiveness of interventions using SMTP.

## Methods

### Construction and validation process of SMSTP

We developed an SMSTP based on indicators developed by our research group [38]. Item and exploratory factor analyses (EFA) were conducted to determine the final scale. We sought to establish the measurement properties of this scale, including reliability, content validity*, *and construct validity. Cronbach’s α and intraclass correlation coefficient (ICC) were used to assess the reliability. Additionally, content and construct validity were used to evaluate the validity of the developed scale.

The SMSTP development and validation process was divided into two phases with a total of five steps.

#### Phase 1: Development of the SMSTP

- Step 1: Construction of the questionnaire items

We developed the questionnaire based on an indicator framework that assessed the effects of TB health promotion on the behaviors of patients with TB. The framework was developed by our research group in a previous study and has been reported elsewhere in further detail [38]. In brief, the indicator framework was constructed after a two-round modified Delphi process conducted by 16 TB experts from thirteen provinces or regions in China. The framework contained 3 domains (“Adherence to treatment,” “Healthy lifestyle,” and “Transmission and prevention”), 8 subdomains (including among others, “Adherence to their medication”), and 14 indicators (including “Percentage of patients who adhered to their medication”) [38]. All items on the scale were described as declarative sentences (e.g., Taking medications consistently). Each item contained a five-level scale for responses: (1) never, (2) only rarely, (3) sometimes, (4) Quite often, and (5) Always.

- Step 2: Item analysis

Before the actual survey, we conducted a pre-survey involving 25 TB patients to evaluate the clarity, a understandability of SMSTP. The questions were then modified based on the results from patients’ suggestions and their feelings regarding the questionnaires. The critical ratio (CR) and item-total correlation (ITC) were calculated for each item to determine whether the item should be removed or retained.

1. Critical value analysis: In accordance with each participant's score per item, scores in the highest 27 percent and the lowest 27 percent were divided into high- and low-score groups, respectively; and an independent t-test was conducted. A statistically significant CR value indicated that the item had satisfactory differentiation power.
2. ITC: If the correlation coefficient was < 0.4 or a p-value < 0.05 was noted, the item was removed [39].

Both statistical and clinical significance were used to decide whether to include or remove an item.

- Step 3: Exploratory Factor Analysis

An EFA was performed for the remaining items using the maximum likelihood method with Promax rotation. Kaiser–Meyer–Olkin (KMO) statistics and Bartlett’s test of sphericity were used to determine the sample adequacy of factoring. KMO values higher than 0.7 were acceptable and values between 0.8 and 0.9 indicated a strong relationship [40]. Items were retained when the following criteria were fulfilled: (1) item factor loading > 0.4 and (2) items were conceptually consistent with their corresponding dimensions. A scree plot and the Kaiser criterion, which suggests keeping dimensions with eigenvalues ≥ 1.0 [41], were used to identify the optimal number of dimensions for further analysis. The first point at which eigenvalues began to level off was considered the maximum dimension that should be extracted.

#### Phase 2: Validation of the SMSTP

- Step 4: Reliability analysis
  Internal consistency and test–retest reliability were assessed to determine the reliability of the SMSTP. Internal consistency was determined by the degree of inter-relatedness among items and was assessed using Cronbach’s alpha [42]. Cronbach’s α values > 0.60 were considered acceptable, and those > 0.7 were considered satisfactory [43, 44]. Fifty participants in SMSTP testing returned after 2 weeks and were assessed for test–retest reliability. The test–retest reliability index was based on the intraclass correlation coefficient (ICC), and the 95% confidence interval was calculated. An ICC ≥ 0.7 indicated good test–retest reliability [45, 46].
- Step 5: Validity
  Content and construct validity of the SMSTP were assessed. Content validity was calculated based on the correlation between each item and dimensions using Pearson’s correlation, with values > 0.8 considered very strong, values between 0.6 and 0.8 considered strong, and values between 0.3 and 0.6 considered moderate [47]. Additionally, confirmatory factor analysis (CFA) was used to evaluate construct validity, which was estimated using multiple fit indices, such as relative chi-square and degrees of freedom (χ^2^/df), comparative fit index (CFI), normed fit index (NFI), incremental fit index (IFI), goodness of fit index (GFI), adjusted goodness of fit index (AGFI), root mean square error of approximation (RMSEA), and standardized root means square residual (SRMR). CFI > 0.90, NFI > 0.90, IFI > 0.90, GFI > 0.95, AGFI > 0.90, and RMSEA and SRMR < 0.06 indicated a good fit (SRMR < 0.08 was acceptable) [48–50]. Convergent and discriminant validity were also analyzed to evaluate construct validity. Convergent validity was evaluated using the average variance extracted (AVE), with an AVE > 0.7 indicating good convergent validity. We evaluated discriminant validity using two criteria: (1) The Fornell-Larcker criterion, which states that the discriminant validity of AVEs is supported by their square root being higher than their inter-construct correlations [51], and (2) the heterotrait–monotrait (HTMT) ratio, in which a value of < 0.85 reflects the presence of discriminant validity [52].

### Data collection

The survey was conducted on a professional online survey platform (Changsha Ranxing Information Technology Co., Ltd.). Participants were sent an electronic link to the questionnaire through WeChat, the most commonly used social media application in China [53] and were invited to complete the survey via mobile phone or computer. We restricted the IP address of each device (mobile phone or computer) to only one survey.

The first page of the online questionnaire contained informed consent details, where participants had to click the “Yes, I consent” option to proceed to the start page of the survey. Participants could pause and continue the questionnaire at any time, and the researchers could view questionnaire responses at any time. One member of the survey team called the participants by telephone after Phase 1 completion and asked if they would be willing to complete the questionnaire again. Fifty participants were willing to redo the questionnaire and received electronic links to the same questionnaire through WeChat two weeks after the initial submission. Participants had to answer all questions in order to submit it.

### Participants

Participants were recruited through the Chongqing Public Health Medical Treatment Center, which is the largest tertiary hospital with grade A status for infectious diseases and the designated TB hospital in Chongqing [54]. Eligibility criteria for participants were as follows: (1) diagnosed with pulmonary TB according to WHO guidelines, (2) aged at least 15 years (participants under the age of 18 years required consent from a parent and/or legal guardian), (3) ability to use WeChat. The exclusion criteria, on the other hand, included: (1) patients with extra-pulmonary TB, (2) patients with a history of cognitive impairment or psychiatric disease, (3) patients who declined participation, and (4) patients unable to use WeChat.

The sample size should be 5–10 times the number of items when developing a questionnaire [55, 56]. For this study, the initial scale contains 20 items, so the estimated sample size is 100–200. This study was divided into two phases: questionnaire construction and its validation, so a sample size of 100–200 was required for each phase. In the first stage of the survey, a total of 231 patients with TB were recruited for the study, in which the responses were used to determine the item analysis and EFA of the SMSTP. In the second stage of the survey, an additional 231 TB patients participated, in which the responses were used for the CFA in assessing the validity and reliability of the SMSTP.

### Data analysis

The Statistical Package for Social Science (SPSS 25.0, IBM Corporation, Armonk, NY, USA) was used to analyze the data. Independent sample t-tests were used to compare patient demographic characteristics in the two phases. CR, item-total correlation, and EFA were used to screen the items. Cronbach’s α and ICC were calculated to assess reliability. The validity was examined using CFA. Statistical significance was set at p < 0.05.

## Results

### Demographic characteristics of the participants

The survey completion rate was 100%. A total of 462 participants were included in this study. More than two-thirds (70.6% in Phase 1 and 67.9% in Phase 2) of the participants were aged 18–39 years. The majority of the participants were male (54.5% in Phase 1 and 57.1% in Phase 2). In both groups, the proportion of rural residents was greater than 60% (66.7% in Phase 1 and 63.6% in Phase 2). Regarding marital status, the proportion of unmarried patients was highest in both groups (64.1% in Phase 1 and 58.4% in Phase 2). Students were the largest proportion in terms of profession (35.1% in Phase 1 and 38.5% in Phase 2). Overall, Phase 1 and Phase 2 participants did not differ significantly in terms of demographic characteristics (Table 1).

**Table 1:** Demographic characteristics of tuberculosis patients in two phases (n = 462)

Demographic characteristics	Total	Phase 1 (n = 231)	Phase 2 (n = 231)	χ^2^	*p*
Gender					
Male	258 (55.8)	126 (54.5)	132 (57.1)	2.794	0.574
Female	204 (44.2)	105 (45.5)	99 (42.9)		
Age					
15–17	32 (6.9)	17 (7.4)	15 (6.5)	2.177	0.674
18–39	320 (69.3)	163 (70.6)	157 (67.9)		
40–59	97 (21.0)	43 (18.6)	54 (23.4)		
≥ 60	13 (2.8)	8 (3.4)	5 (2.2)		
Ethnicity					
Han race	335 (72.5)	159 (68.8)	176 (76.2)	3.318	0.076
Others	127 (27.5)	72 (31.2)	55 (23.8)		
Residence					
Urban	161 (34.8)	77 (33.3)	84 (36.4)	0.467	0.494
Rural	301 (65.2)	154 (66.7)	147 (63.6)		
Registered information					
Resident	411 (89.0)	200 (86.6)	211 (91.3)	2.667	0.102
Migrant	51 (11.0)	31 (13.4)	20 (8.7)		
Marital status					
Unmarried	283 (61.3)	148 (64.1)	135 (58.4)	1.609	0.477
Married	167 (36.1)	77 (33.3)	90 (39.0)		
Divorced/Widowed	12 (2.6)	6 (2.6)	6 (2.6)		
Education					
Primary and below	67 (14.5)	35 (15.2)	32 (13.9)	0.339	0.844
Junior middle school	129 (27.9)	66 (28.6)	63 (27.2)		
High school and above	266 (57.6)	130 (56.3)	136 (58.9)		
Occupation					
Staff/Cadre/Retire	106 (22.9)	49 (21.2)	57 (24.7)	4.954	0.138
Self-employed	61 (13.2)	29 (12.6)	32 (13.8)		
Farmer/Migrant worker	107 (23.2)	65 (28.1)	42 (18.2)		
Student	170 (36.8)	81 (35.1)	89 (38.5)		
Others	18 (3.9)	7 (3.0)	11 (4.8)		

### Item analysis

According to the indicator framework, we included 20 items across the three dimensions (“adherence to treatment behavior,” “supportive behavior,” and “transmission prevention behavior”) in the first draft of the SMSTP (Table 2). Each item was rated on a 5-point Likert scale (1 = never, 2 = rarely, 3 = sometimes, 4 = quite often, and 5 = always), with a higher score indicating a higher level of self-management. After CR and ITC calculations, the means of all items ranged from 2.79 to 4.13. The ITC were all statistically significant, except for items 1 and 3. Given the results of statistical analysis and clinical significance, we finally deleted items 1 and 3. Eighteen items were ultimately retained in the SMSTP (Table 3).

**Table 2:** The first draft of SMSTP

Dimensions	Item contents (brief description in English)	Never	Only rarely	Sometimes	Quite often	Always
Adherence to treatment behavior	Item1:Medication-taking record card filled					
	Item2: Taking medications following prescription					
	Item3:Taking medications when going out					
	Item4: Keeping drugs correctly					
	Item5:Addressing adverse effects of medications correctly					
	Item6: Taking medications consistently					
	Item7: Regular following-up sputum microscopy					
Supportive behavior	Item8:Abstaining from smoking					
	Item9:Abstaining from alcohol drinking					
	Item10:Keeping adequate sleep					
	Item11: Avoiding overexertion					
	Item12:Keeping proper exercise					
	Item13: Keeping adequate nutrition					
Transmission prevention behavior	Item14:Not spitting indiscriminately					
	Item15: Reducing frequency of presence in public					
	Item16: Wearing respirator in public during in infective phrase of disease					
	Item17:Disposing sputum with safe method					
	Item18:Covering face when sneezing/cough/speaking loudly					
	Item19: Informing contact of TB status					
	Item20: Ventilating their room					

*SMSTP* self-management scale for TB patients

**Table 3:** Results of item analysis of SMSTP (n = 231)

Dimension	Item (brief description in English)	Mean (SD)	Critical value	Item-total correlation coefficient
Adherence to treatment behavior	Item 1: Medication-taking record card filled	3.97 ± 1.38	0.823	0.060
	Item 2: Taking medications following prescription	4.00 ± 1.29	14.600**	0.676**
	Item 3: Taking medications when going out	3.07 ± 1.39	0.372	0.035
	Item 4: Keeping drugs correctly	3.21 ± 1.46	10.648**	0.603**
	Item 5: Addressing adverse effects of medications correctly	2.94 ± 1.40	12.589**	0.602**
	Item 6: Taking medications consistently	3.14 ± 1.49	13.746**	0.616**
	Item 7: Regularly following-up sputum	2.99 ± 1.45	14.952**	0.661**
Supportive behavior	Item 8: Abstaining from smoking	2.95 ± 1.14	11.471**	0.600**
	Item 9: Abstaining from alcohol drinking	3.16 ± 1.23	10.190**	0.575**
	Item 10: Maintaining adequate sleep	2.90 ± 1.05	10.332**	0.578**
	Item 11: Avoiding overexertion	3.17 ± 1.25	12.533**	0.643**
	Item 12: Keeping proper exercise	2.79 ± 0.91	11.522**	0.620**
	Item 13: Maintaining adequate nutrition	3.19 ± 1.30	11.406**	0.624**
Transmission prevention behavior	Item 14: Not spitting indiscriminately	2.82 ± 0.98	10.505**	0.574**
	Item 15: Reducing frequency of presence in public	3.69 ± 1.05	11.428**	0.735**
	Item 16: Wearing respirator in public during infective phase of disease	4.13 ± 1.21	5.386**	0.407**
	Item 17: Disposing sputum with safe method	3.39 ± 1.21	10.573**	0.653**
	Item 18: Covering face when sneezing/coughing/speaking loudly	3.38 ± 1.17	11.425**	0.685**
	Item 19: Informing contact of TB status	3.24 ± 1.16	9.520**	0.599**
	Item 20: Ventilating their room	3.32 ± 1.16	10.046**	0.595**

*SMSTP* self-management scale for TB patients; **P < 0.001

### Exploratory factor analysis

**Fig. 1:**
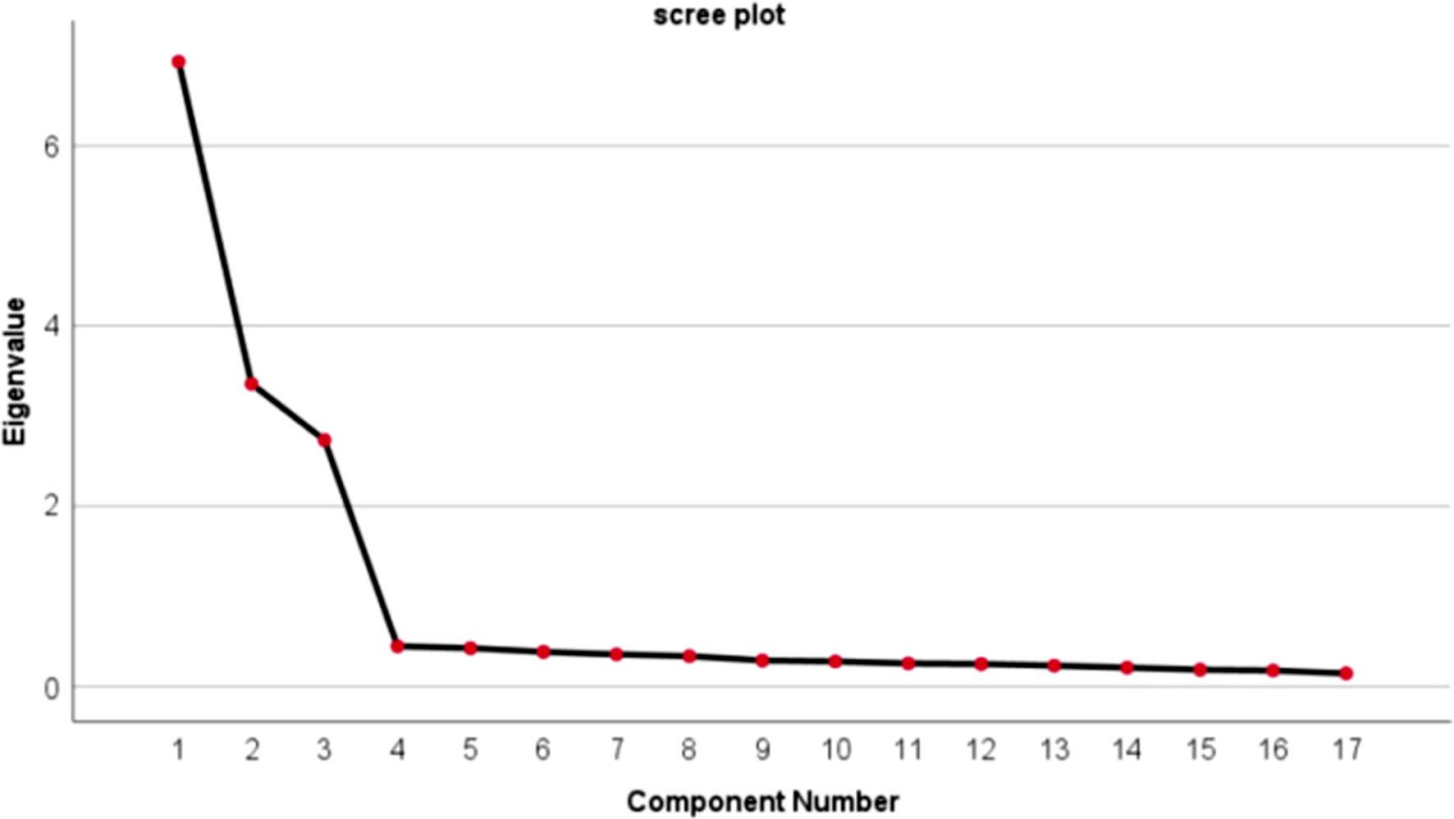
Scree plot of principal component factor analysis. This figure shows the eigenvalue plot for the SMSTP ordered from largest to smallest. The Y axis represents eigenvalues for the dimensions of the SMSTP, and the X axis represents the different dimensions of the SMSTP

The KMO value was 0.97, and Bartlett’s test value was 8882.47 (p < 0.001), which indicated that the data could be used for factor analysis. Four dimensions were extracted in the first EFA, with an eigenvalue greater than one, which accounted for 67.74% of the total variance. Item 14 was dropped with a rotated factor loading of lower than 0.4. After performing the second EFA, three dimensions were extracted with eigenvalues greater than 1, all of which were also supported by the scree plot (Fig. 1). These three dimensions accounted for 76.60% of the variance. In each dimension, all items had rotation factor loadings greater than 0.8 and no item was loaded on more than one dimension. After two rounds of EFA, the final version of the SMSTP was developed, which included three dimensions and 17 items. The number of dimensions extracted by EFA and the items contained in each dimension were consistent with the initial questionnaire. Table 4 shows the rotated factor loadings of the final version of the SMSTP.

**Table 4:** The results of rotated factor matrix for final exploratory factor analysis

Dimension\Item (brief description in English)	Factor loadings			Eigenvalue	Explained Variance (%)
	1	2	3		
Dimension 1 (Transmission prevention behavior)					
Item 15: Reducing frequency of presence in public	**0.819**	0.076	0.173	6.932	26.916
Item 16: Wearing respirator in public during in infective phase of disease	**0.847**	0.179	0.231		
Item 17: Disposing sputum with safe method	**0.859**	0.109	0.157		
Item 18: Covering face when sneezing/cough/speaking loudly	**0.856**	0.129	0.192		
Item 19: Informing contact of TB status	**0.867**	0.099	0.074		
Item 20: Ventilating their room	**0.865**	0.100	0.039		
Dimension 2 (Supportive behavior)					
Item 8: Abstaining from smoking	0.114	**0.859**	0.107	3.357	26.761
Item 9: Abstaining from alcohol drinking	0.062	**0.881**	0.070		
Item 10: Maintaining adequate sleep	0.038	**0.843**	0.139		
Item 11: Avoiding overexertion	0.197	**0.851**	0.089		
Item 12: Keeping proper exercise	0.117	**0.864**	0.064		
Item 13: Maintaining adequate nutrition	0.154	**0.837**	0.171		
Dimension 3 (Adherence to treatment behavior)					
Item 2: Taking medications following prescription	0.191	0.125	**0.889**	2.734	22.929
Item 4: Keeping drugs correctly	0.102	0.101	**0.856**		
Item 5: Addressing adverse effects of medications correctly	0.103	0.142	**0.841**		
Item 6: Taking medications consistently	0.142	0.088	**0.857**		
Item 7: Regularly following-up sputum microscopy	0.211	0.115	**0.840**		

Bold values: items loading significant to a factor

The first dimension, “transmission prevention behavior,” accounted for 26.91% of the total variance. This dimension included behaviors and activities that reduced the spread of *Mycobacterium tuberculosis*, including reducing the frequency of exposure to public places, wearing masks, covering the mouth and nose when coughing or sneezing, disposing of sputum properly, informing family and friends about TB infections, and urging them to screen for TB. The second dimension, “supportive behavior,” consisted of 6 items and accounted for 26.76% of the total variance. These items were related to lifestyle choices, such as smoking, drinking, exercising, sleeping, eating, and avoiding fatigue. Finally, the third dimension, “adherence to treatment behavior,” accounted for 22.92% of the total variance and included key behaviors related to TB treatment, such as medication adherence, methods of administration, storage of medications, reexaminations, and treatment of side effects.

### Reliability

Cronbach’s α for the 17-item scale was 0.905 and varied from 0.925 to 0.936 for each dimension. The test–retest ICC for the total scale was 0.897, while it was 0.885, 0.834, and 0.814 for dimensions 1, 2, and 3, respectively (n = 50, *p* < 0.001) (Table 5).

**Table 5:** Reliability and convergent validity of SMSTP

	Cronbach Alpha	ICC (95%CI)	AVE
Dimension 1: Transmission prevention behavior	0.936	0.814 (0.693–0.890)	0.715
Dimension 2: Supportive behavior	0.933	0.834 (0.807–0.933)	0.711
Dimension 3: Adherence to treatment behavior	0.925	0.885 (0.807–0.933)	0.709
Total	0.905	0.897 (0.825–0.940)	

*SMSTP* self-management scale for TB patients, *ICC* intraclass correlation coefficient, *AVE* average variance extracted

### Validity

#### Content validity

According to Pearson’s correlation analysis, the scores for each item were strongly correlated with the dimension to which they belonged (r = 0.849–0.915, *p* < 0.01). There were weak, positive correlations between the dimensions (r = 0.267–0.344, *p* < 0.01). Additionally, the correlation of each item with its contributive dimension was higher than that with other dimensions (Table 6).

**Table 6:** Pearson correlation analysis between items and dimensions of SMSTP (r)

Dimension	Item	Transmission prevention behavior	Supportive behavior	Adherence to treatment behavior
Transmission prevention behavior		1	0.275**	0.344**
	Item 15: Reducing frequency of presence in public	0.204**	0.849**	0.315**
	Item 16: Wearing respirator in public during infective phase of disease	0.312**	0.887**	0.388**
	Item 17: Disposing sputum with safe method	0.237**	0.880**	0.311**
	Item 18: Covering face when sneezing/coughing/speaking loudly	0.262**	0.885**	0.347**
	Item 19: Informing contact of TB status	0.220**	0.870**	0.238**
	Item 20: Ventilating their room	0.216**	0.863**	0.207**
Supportive behavior		0.275**	1	0.267**
	Item 8: Abstaining from smoking	0.878**	0.241**	0.236**
	Item 9: Abstaining from alcohol drinking	0.887**	0.190**	0.195**
	Item 10: Maintaining adequate sleep	0.849**	0.175**	0.249**
	Item 11: Avoiding overexertion	0.882**	0.312**	0.233**
	Item 12: Keeping proper exercise	0.865**	0.236**	0.198**
	Item 13: Maintaining adequate nutrition	0.855**	0.286**	0.297**
Adherence to treatment behavior		0.344**	0.267**	1
	Item 2: Taking medications following prescription	0.346**	0.346**	0.915**
	Item 4: Keeping drugs correctly	0.256**	0.256**	0.868**
	Item 5: Addressing adverse effects of medications correctly	0.261**	0.261**	0.857**
	Item 6: Taking medications consistently	0.292**	0.292**	0.873**
	Item 7: Regularly following-up sputum	0.355**	0.355**	0.873**

*SMSTP* self-management scale for TB patients, *r* Pearson’s correlation coefficient; **P < 0.01

#### Construct validity

Fit indices, convergent validity, and discriminant validity were evaluated using CFA to determine construct validity. The results of EFA indicated that all three dimensions had characteristic roots > 1. CFA was performed to examine whether the three-dimension model extracted by EFA could explain the pattern of relationships among the items. The fit indices for the final 17-item model were χ^2^/df = 1.579, CFI = 0.979, NFI = 0.944, IFI = 0.979, GFI = 0.916, AGFI = 0.889, RMSEA = 0.059 (90% CI: 0.036–0.063), and SRMR = 0.041. Moreover, convergent validity was evaluated through AVE*.* As shown in Table 5, all the AVE values of the three dimensions were higher than 0.5. We examined the discriminant validity using the Fornell–Larcker criterion and HTMT ratio. The square root values of the AVE for each dimension were greater than the correlations between this dimension and other dimensions (Table 7). In addition, all HTMT values were below 0.85.

**Table 7:** Fornell–Larcker criterion and HTMT ratio values

	Dimension1: Transmission prevention behavior	Dimension2: Supportive behavior	Dimension3: Adherence to treatment behavior
Dimension1: Transmission prevention behavior	**0.845**	0.297	0.377
Dimension2: Supportive behavior	0.274	**0.843**	0.297
Dimension3: Adherence to treatment behavior	0.351	0.275	**0.842**

*HTMT* Heterotrait–Monotrait, Fornell–Larcker’s criteria = Bold values on the diagonal are the square root of AVE; values above the diagonal are HTMT values, while those below are the correlations between dimensions

## Discussion

In this study, we developed SMSTP to measure SMTP and evaluate the effectiveness of interventions. The SMSTP consisted of 17 items in three dimensions, which focused primarily on the behavioral aspects of SMTP. Previous studies reported psychological dimension in instrument of self-management which evaluated the associations between psychological dimension and partnership with healthcare professionals, and self-management [57–59]. Even though, psychological and partnership with healthcare professionals are important factors for SMTP behavior’s adaption or change, the SMSTP focused on evaluating the level or change of behaviors related to SMTP. According to the results, the SMSTP demonstrated good reliability and validity and could be used to assess and develop SMTP interventions. Future research should consider other dimensions of self-management, such as psychological dimension and the dimension of the relationship with professionals.

Although there is no uniform definition, self-management is considered an important part of managing chronic disease, with the aim of illness prevention and promoting health [60]. Health Promotion is defined as the process of enabling people to increase control over and improve their health [61]. Thus, self-management can be considered an important goal of health promotion. In our previous study, we constructed an indicator framework for TB health promotion. The framework contains not only treatment related behaviors but also health-related behaviors that contribute curing TB, such as improving nutrition, avoiding smoking, and prohibiting alcohol consumption. In addition, from the perspective of public health, the framework also includes indicators of behaviors that prevent TB. If TB patients possess these abilities, they will have the ability to manage TB and promote their health, which is self-management. Therefore, in this study, we developed the SMSTP based on the framework.

It can be concluded based on factor analysis that neither Item 1 nor Item 3 in the initial scale reached statistical significance. In terms of clinical significance, Item 1 (Medication-taking record card filled), Item 3 (Taking medications when going out), and Item 6 (Taking medications consistently), all investigated a patient's adherence to medication. Item 1 was an external means to help a patient adhere to medication, Item 3 was a demonstration of medication adherence, and Item 6 could directly assess whether a patient adhered to medication. Considering the results of statistical analysis and clinical significance, we finally deleted Items 1 and 3.

After two-round EFA, we developed the SMSTP with 17 items to assess the self-management levels of patients with TB. Three dimensions were extracted from the 17 items that accounted for 76.60% of the total variance. Furthermore, later internal consistency evaluations showed that Cronbach's α exceeded 0.9 for the total scale and each of the dimensions, indicating that the scale had high internal consistency and that no additional adjustments to the SMSTP were required.

Notably, the most influential dimension was “transmission prevention behavior” followed by “supportive behavior” and “adherence to treatment behavior.” This finding was inconsistent with existing empirical evidence [62, 63]. Both the DOTS strategy recommended by the WHO [64] and the vast majority of studies [25, 65, 66] suggest that treatment adherence is an important determinant of TB control. TB can spread by air, and each patient with active TB can infect an average of 10–15 individuals each year [67]. Accordingly, transmission prevention behaviors among patients with TB may be one of the key measures in reducing community transmission. Moreover, the National Health Commission of China’s Technical Specifications for Tuberculosis Prevention and Control [68] highlighted the importance of prevention. Our results also showed that “transmission prevention behavior” could more likely predict improved SMTP than “adherence to treatment behavior,” which may suggest that TB health promotion should focus more on these behaviors.

Several studies have demonstrated that self-management performance is primarily influenced by a patient's characteristics and lifestyle choices [69]. The EFA results revealed that “supportive behavior,” accounting for 69% of the total variance of TB self-management, was also higher than “adherence to treatment behavior.” Smoking, alcohol consumption, and poor nutritional status are known to be associated with poor treatment outcomes in patients with TB [70, 71]. Furthermore, lifestyle interventions have become increasingly popular strategies for chronic disease management [72]. Hence, lifestyle modifications should be considered an important component of TB self-management interventions.

Cronbach’s α values of the SMSTP were greater than 0.9 for all dimensions and the total scale, indicating a high level of internal consistency and reliability. Test–retest reliability showed that the same rater's scores were consistently measured over time. Typically, the ideal interval between the test and retest is 2–4 weeks [73–75]. Considering that test–retest reliability with a time interval greater than 2 weeks may be affected by the fluctuation process of the behavior itself, the test–retest reliability was tested for a time interval of two weeks*.* The ICC values of the three dimensions in the SMSTP were all > 0.7, greater than the standard threshold [76]. This indicated that the measurement time had little influence on the reliability of this scale, and that it had strong time flexibility and stability in assessing SMTP. In conclusion, our findings indicate that the SMSTP had good overall reliability.

The SMSTP was found to have excellent content validity in terms of questionable relevance. Correlation analysis showed high correlations (> 0.8) with the dimensions and only moderate or weak correlations in the other dimensions. In addition, correlations between the dimensions were weak. These findings imply that close associations between items and dimensions could clarify the practical sense of the dimension to which they belonged. Each dimension and the items they contained were also distinguishable from the other dimensions. Therefore, the three dimensions could be used separately to assess the different components of self-management depending on the different needs of assessment.

EFA results revealed that the KMO test (KMO = 0.970) and Bartlett’s test of sphericity were ideal for factor analysis. Moreover, the cumulative contribution rate of this study was over 76%, which indicated that the SMSTP could adequately measure the level of SMTP and confirmed good construct validity of the questionnaire. CFA was also used to verify the construct validity of the scale. All CFI, TLI, NFI, IFI, and GFI values were above 0.9, and the χ^2^/df values were within the ranges suggested by Wheaton [77]. The RMSEA was less than 0.08, and the RMR was less than 0.05, both meeting the thresholds recommended by Browne and Cudeck [78]. Only one index, AGFI, was 0.889 and did not reach the standards that would indicate an acceptable model fit. This index, however, is likely to be underestimated when the sample size is less than 300 [79]. Overall, the CFA model fit indices of the SMSTP were acceptable, but less than perfect.

Additionally, convergent and discriminant validity were assessed to evaluate construct validity [80]. A high level of convergent validity can assist researchers in understanding how the constructs of the three measures are interconnected both theoretically and practically [81]. To determine convergent validity, we needed to know how well the latent construct could explain the variance of each indicator, which was assessed through AVE [82]. AVE represented the mean value of the commonality of the indicators of a certain construct [83]. Our results indicated that the AVE of all three dimensions of the SMSTP was greater than the critical value of 0.5, demonstrating that the amount of variance between dimensions and corresponding indicators exceeded that caused by measurement errors [83]. Thus, the convergent validity of the SMSTP was established.

Furthermore, we assessed discriminant validity using the Fornell–Larcker criterion and HTMT. First, the square root of the AVE for each dimension was greater than the correlation between it and the other dimensions, which met the Fornell–Larcker criterion [84]. Second, the HTMT values of the correlations were used to assess the discriminant validity. The HTMT is determined by comparing the average correlations of indicators across constructs, which measure different constructs to those indicative of the same construct [85]. According to the criteria of Henseler et al. [86], the HTMT values should be less than 0.85. Results from this study revealed that all HTMT values were far below the conservative 0.85 upper bound, illustrating that the SMSTP constructs were separable from each other. Overall, the results of both the Fornell–Larcker criterion and HTMT implied that the scale had good discriminative validity.

### Strengths and limitations

SMTP is a patient-centered TB case management model. This study developed and validated the SMSTP as a new instrument for assessing SMTP, which could be used in future research and practice. The results of an SMSTP assessment may lead to the development of target interventions. The SMSTP may also be used to evaluate the effects of interventions on SMTP. In addition, there are still some limits in this study: First, in that participants were recruited from one province (Chongqing) in China, and the sample population may not represent the entire population of patients with TB in China. Further studies should be conducted in different regions to determine the efficacy of the SMSTP. Second, self-management involves multiple aspects of ability. The SMSTP focused primarily on the dimensions of behavioral aspects, including clinical management, establishing a healthy lifestyle, and preventing the spread of disease. Another limitation of our study is the lack of application of scale. This is an ongoing study and we have started the implementation research in Chongqing, but it has not completed, and we will go on this research in the future.

## Conclusion

This study developed and validated SMSTP which consisted of 17 items in three dimensions: adherence to treatment behavior (5 items), supportive behavior (6 items), and transmission prevention behavior (6 items). Each item was rated on a 5-point Likert scale, and where higher scores indicated a higher self-management. SMSTPS will be used to design intervention strategies for SMTP and evaluate the effectiveness of interventions on SMTP.

## Data Availability

All data generated or analysed during this study are included in this published article.

## Abbreviations

AGFI: Adjusted goodness of fit index
CFA: Confirmatory factor analysis
CFI: Comparative fit index
DOT: Directly observed therapy
DOTS: Directly observed therapy, short-course
EFA: Exploratory factor analysis
GFI: Goodness of fit index
HTMT: Heterotrait–Monotrait
ICC: Intraclass correlation coefficient
IFI: Incremental fit index
ITC: Item-total correlation
KMO: Kaiser–Meyer–Olkin
NFI: Normed fit index
RMSEA: Root mean square error of approximation
SRMR: Standardized root mean square residual
SMTP: Self-management of tuberculosis patients
SMSTP: Self-management scale of tuberculosis patients
TB: Tuberculosis
WHO: World Health Organization
